# Performance of Rod-Shaped Ce Metal–Organic Frameworks for Defluoridation

**DOI:** 10.3390/molecules28083492

**Published:** 2023-04-15

**Authors:** Jiangyan Song, Weisen Yang, Xiaoshuai Han, Shaohua Jiang, Chunmei Zhang, Wenbin Pan, Shaoju Jian, Jiapeng Hu

**Affiliations:** 1 College of Environment and Safety Engineering, Fuzhou University, Fuzhou 350001, China; sjya2689@163.com (J.S.);; 2 Key Laboratory of Green Chemical Technology of Fujian Province University, Fujian Provincial Key Laboratory of Eco-Industrial Green Technology, Wuyi University, Wuyishan 354300, China; yangweisen@126.com (W.Y.); hxs141424@163.com (X.H.); shaohua.jiang@njfu.edu.cn (S.J.); 3 Jiangsu Co-Innovation Center of Efficient Processing and Utilization of Forest Resources, International Innovation Center for Forest Chemicals and Materials, College of Materials Science and Engineering, Nanjing Forestry University, Nanjing 210037, China; 4 Institute of Materials Science and Devices, School of Materials Science and Engineering, Suzhou University of Science and Technology, Suzhou 215009, China

**Keywords:** Ce-H3TATAB-MOFs, fluoride, adsorption, metal–organic frameworks

## Abstract

The performance of a Ce(III)-4,4′,4″-((1,3,5-triazine-2,4,6-triyl) tris (azanediyl)) tribenzoic acid–organic framework (Ce-H3TATAB-MOFs) for capturing excess fluoride in aqueous solutions and its subsequent defluoridation was investigated in depth. The optimal sorption capacity was obtained with a metal/organic ligand molar ratio of 1:1. The morphological characteristics, crystalline shape, functional groups, and pore structure of the material were analyzed via SEM, XRD, FTIR, XPS, and N_2_ adsorption–desorption experiments, and the thermodynamics, kinetics, and adsorption mechanism were elucidated. The influence of pH and co-existing ions for defluoridation performance were also sought. The results show that Ce-H3TATAB-MOFs is a mesoporous material with good crystallinity, and that quasi-second kinetic and Langmuir models can describe the sorption kinetics and thermodynamics well, demonstrating that the entire sorption process is a monolayer-governed chemisorption. The Langmuir maximum sorption capacity was 129.7 mg g^−1^ at 318 K (pH = 4). The adsorption mechanism involves ligand exchange, electrostatic interaction, and surface complexation. The best removal effect was reached at pH 4, and a removal effectiveness of 76.57% was obtained under strongly alkaline conditions (pH 10), indicating that the adsorbent has a wide range of applications. Ionic interference experiments showed that the presence of PO_4_^3−^ and H_2_PO_4_^−^ in water have an inhibitory effect on defluoridation, whereas SO_4_^2−^, Cl^−^, CO_3_^2−^, and NO_3_^−^ are conducive to the adsorption of fluoride due to the ionic effect.

## 1. Introduction

With the boom of the economy and industrialization, water pollution and water resource problems have become pressing concerns. In particular, water fluorine pollution is currently considered a severe water quality issue in the world [1], and the development of effective and economical methods to tackle this stubborn problem is required. Fluorine pollution refers to environmental pollution caused by excess fluorine and its compounds, either due to human activities or natural processes, such as industrial effluents using fluorinated materials, weathering of mineral-rich ground, volcanic activity, and fluoride (F^−^) release from marine aerosols [2,3], causing a serious threat to animals, plants, and humans. Fluoride is one of the indispensable trace elements for humankind and a major component of bones and teeth, hence, it is irreplaceable and yet excessive or insufficient ingestion can pose adverse influences on human health [4]. Contact with a high level of F^−^ for long periods via drinking water, air, or food can lead to a succession of health problems, such as fluorosis, dental fluorosis, neurological disorders, Alzheimer’s disease, and inhibition of the breakdown of enzymes in the body, affecting normal metabolism [5,6,7]. Consequently, the F^−^ content in drinking water must be controlled in accordance with the World Health Organization (WHO) threshold of 1.5 mg L^−1^.

Conventional technologies to tackle the issue of fluorine pollution are adsorption, precipitation, membrane separation, ion exchange, and electrochemical methods [8,9]. Unfortunately, the high cost or limited performance of some of these technologies hinder their practical application. Moreover, adsorption is a widely used technology for fluoride elimination, owing to its outstanding performance, low preparation and operational costs, ease of operation, and lack of secondary pollution [10,11,12]. The main factors determining the performance in F^−^ removal via adsorption are the choice of adsorbent and the operating conditions. Specifically, an appropriate adsorbent should possess high adsorption efficiency, a fast adsorption rate, excellent water stability and mechanical strength, high selectivity for fluoride, and easy production; furthermore, it should fulfil economic and environmental requirements. Several types of adsorbents have been exploited for F^−^ elimination, such as zeolite, hydroxyapatite, biochar, bentonite, metal–organic frameworks (MOFs), and layered double hydroxides [13,14,15,16,17,18]. However, most of these adsorbents suffer from low adsorption capacity, poor selectivity, and high treatment costs; thus, the development of superior adsorbents still constitutes an important yet challenging task.

Of particular interest in this context are MOFs, which are one-, two-, three-, and four-dimensional organic–inorganic hybridized reticular polymeric structures constructed via self-assembly of metal ions/clusters and organic ligands [19,20]. The type, topology, and physicochemical properties largely vary on the category of the metal, the use of organic ligands, and the composition method [21]. Generally, MOFs feature tunable structures, high porosity, easy functionalization, and large specific surface areas, which enable their utilization in a wide range of applications, not only in wastewater treatment but also in the fields of drug transport, sensing, energy storage, and catalysis [22,23,24,25,26]. The porosity of MOFs facilitates the diffusion of pollutants to the binding sites leading to rapid adsorption saturation, and their chemical functional groups can be easily adjusted to facilitate adsorption [21]. The employment of MOFs for the elimination of excess F^−^ in aqueous solutions has attracted intense research attention. However, achieving high removal efficiency for specific pollutants is difficult because their adsorption properties depend on various parameters, including the specific surface area, pore size, morphology, hydrogen bonding, and hydrophobic–hydrophilic effects. In order to surmount these drawbacks, the efficiency of target compound identification can be enhanced by precise molecular design; structure tunability; the usage of diverse organic ligands; the insertion of nanocarrier substances; and the modulation of surface charge, polarity, and pore size [27]. Although most MOFs can achieve F^−^ removal, their performance is still insufficient. Accordingly, improving the performance of MOFs via functionalization and modification has become a prevalent research subject. MOFs of different dimensions exhibit different adsorption effects, and the optimization, modification, and introduction of defects can enhance the sorption performance [28]. Currently, the most common MOFs used for defluoridation are based on rare-earth metals, transition metal ions, or magnetic metal ions, used to serve as a metal source, and terephthalic acid or homotrizoic acid as the ligands. Rare-earth metals such as La and Ce possess a high affinity for various anionic pollutants present in the environment owing to their unique chemical properties. Our group prepared Ce-La-MOFs from composite materials with rare-earth metals La and Ce, and applied it in the field of fluoride removal. This study shows that Ce-La-MOFs have an excellent performance on the removal of excess fluoride ions in water [29]. He et al. [30] found that Ce(III) has great potential for F^−^ and phosphorus removal and fabricated a collection of Ce(III)-terephthalate MOFs with linker defects, achieving excellent F^−^ removal with a maximum F^−^ sorption capacity (128 mg g^−1^). The adsorption process was proposed to follow a ligand exchange mechanism that depended on the concentration of F^−^ around the Ce(III) active centers. In the present work, a Ce-H3TATAB-MOFs with excellent performance for F^−^ removal was fabricated via hydrothermal synthesis using Ce as the metal and 4,4′,4″-((1,3,5-triazine-2,4,6-triyl) tris (azanediyl)) tribenzoic acid (H3TATAB) as the ligand, which has not been used to date to prepare MOFs for F^−^ removal. Intermittent adsorption trials were carried out to ensure the optimal sorption conditions, and the impact of the reaction time, initial F^−^ concentration, solution pH, and co-existing anions on the sorption performance was studied. Additionally, the adsorption mechanism was explored deeply using kinetic and thermodynamic studies and material characterization.

## 2. Results and Discussion

The hydrothermal reaction of the 4,4′,4″-((1,3,5-triazine-2,4,6-triyl) tris (azanediyl)) tribenzoic acid (H3TATAB) ligand with the metal Ce in DMF/H_2_O mixture yielded a rod-shaped MOFs, namely Ce-H3TATB-MOFs, as shown in Section 3.

### 2.1. Influence of the Molar Ratio of Precursors

**Figure 1:**
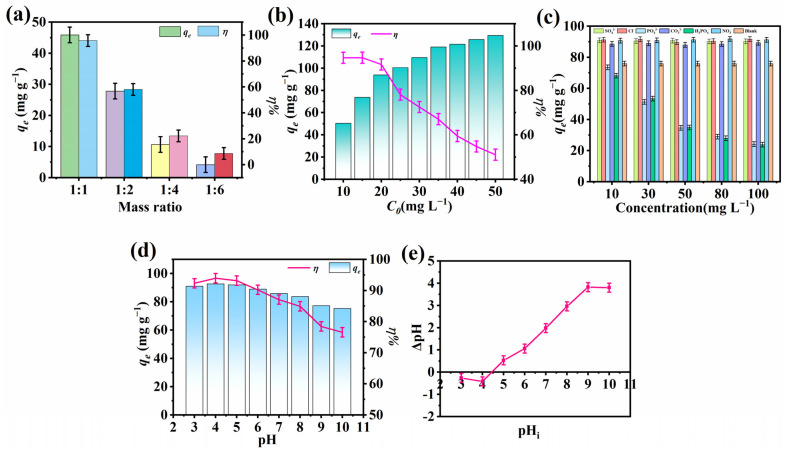
Effect on fluoride removal of (**a**) molar ratio of precursors, (**b**) initial fluoride concentration, (**c**) co-existing ions, (**d**) pH, (**e**) analysis of the zero-point charge (pH_zpc_) for fluoride removal.

The effect of the molar ratio of Ce(NO_3_)_3_·6H_2_O to H3TATAB on F^−^ removal was discussed, and the upshots are shown in Figure 1a. Ce(NO_3_)_3_·6H_2_O and H3TATAB were weighed at molar ratios of 1:1, 1:2, 1:4, and 1:6 and placed in four polyethylene bottles containing 2.5 mL anhydrous ethanol, 2.5 mL DMF, 1 mL water, and 0.024 mL ethylenediamine, respectively. Then, 0.4 mL of concentrated HNO_3_ was added, and the admixture was allowed to react at 100 °C for 72 h. Once cooled to room temperature, the admixture was washed with anhydrous ethanol, then settled, filtered, and dried, and the adsorption capacity (*q_e_*) and removal efficiency (*η*) were measured. Both *q_e_* and *η* were found to gradually decrease with an increasing molar ratio. Therefore, the optimum molar ratio was determined to be 1:1, and the material prepared under this condition was employed for subsequent adsorption experiments.

### 2.2. Influence of Initial F^−^ Concentration

The influence of different initial F^−^ concentrations on F^−^ elimination is shown in Figure 1b. Under the conditions of pH 4, 25 °C, and 0.01 g of Ce-H3TATAB-MOFs, the sorption capacity gradually increased with an increasing initial F^−^ concentration because at the solid/liquid boundary, significant F^−^ concentrations contribute to the energy driving force to overcome the mass transfer barrier in aqueous solutions, causing rapid movement of ions [31] that fully inhabit the active sites of the sorbent, increasing the adsorption capacity. By increasing the initial concentration to 35–50 mg L^−1^, the adsorption capacity changed only slightly, indicating that saturation was gradually attained. From the perspective of the opposite side, the fluoride ion removal efficiency exhibited a decreasing trend because the adsorption sites were limited and became fully occupied when F^−^ ions were initially adsorbed onto the adsorbent, hindering the sorption of the remaining F^−^ [32].

### 2.3. Effect of Co-Existing Ions

The concomitance of multiple ions in water can exert an enhancing effect or an inhibiting effect on F^−^ adsorption. Herein, the impact of SO_4_^2−^, Cl^−^, PO_4_^3−^, CO_3_^2−^, H_2_PO_4_^−^, and NO_3_^−^ on F^−^ removal was explored (Figure 1c), where the concentrations of these anions were 10 mg L^−1^, 30 mg L^−1^, 50 mg L^−1^, 80 mg L^−1^, and 100 mg L^−1^, respectively. The pH was regulated to 4, the Ce-H3TATAB-MOFs was added (0.01 g), and the compound was shaken at 25 °C for 12 h. Subsequently, after allowing the reaction to settle, the mixture was filtered and the F^−^ concentration was measured to calculate the adsorption capacity and evaluate the impact of co-existing ions. Interestingly, the existence of SO_4_^2−^, Cl^−^, CO_3_^2−^, and NO_3_^−^ enhanced the effect of fluoride removal, which increased to 90.86 mg g^−1^, 91.22 mg g^−1^, 88.54 mg g^−1^, and 90.69 mg g^−1^, respectively, at a concentration of 10 mg L^−1^ of these ions. Contrarily, PO_4_^3−^ and H_2_PO_4_^−^ exerted a negative effect, inhibiting F^−^ adsorption by Ce-H3TATAB-MOFs. The reason for this phenomenon may be active site competition of the sorbent surface, which could result in the emergence of inner sphere Ce–P complexes through ligand exchange [30].

### 2.4. Effect of pH

Another crucial factor affecting the removal of F^−^ is the solution pH. The sorption of F^−^ on Ce-H3TATAB-MOFs was investigated with the pH of 3–10. The pH was regulated by HCl and NaOH (0.1 mol L^−1^). Other parameters were kept constant at the optimal values, and the experimental result of the influence of pH for defluoridation is shown in Figure 1d. A higher adsorption capacity and removal efficiency for fluoride were achieved with acidic conditions, particularly at pH 4. Then, after reaching its maximum value, the removal effect gradually decreased as the pH increased. This may owe to the competition effect between F^−^ and OH^−^ under alkaline conditions [33], which is restricted under acidic conditions because depletion of OH^−^ occurs at high proton concentrations, promoting the interaction between F^−^ and the adsorbent [34]. Moreover, the increased negativity of the adsorbent surface enlarges the repulsion between the adsorbent and F^−^ under alkaline conditions, decreasing the removal effect [35]. Nevertheless, the sorption performance of Ce-H3TATAB-MOFs was acceptable in a wide pH range, even at pH 10, with the sorption capacity and removal efficiency reaching 75.3 mg g^−1^ and 76.57%.

The zero-point charge (pH_zpc_) was measured via the pH drift method [36]. In a 50 mL volumetric flask, 50 mL NaCl solution (0.1 mol L^−1^) was measured, the pH was set at 3–10 using NaOH or HCl, 0.01 g of Ce-H3TATAB-MOFs was added, and the reaction was shaken with the temperature and time of 25 °C and 12 h, respectively. After that, the mixture was allowed to precipitate, was filtered, and the pH value of the filtrate was determined. As shown in Figure 1e, the difference between the pH after the reaction and the initial pH (ΔpH) was plotted against the initial pH. The point of pH value at which ΔpH was zero is the pH_zpc_ of Ce-H3TATAB-MOFs [37], which was found to be 4.45. Above this point, the sorption capacity decreases because of the existence of a certain amount of OH^−^, which competes with the active sites of F^−^, resulting in a lower adsorption capacity for F^−^.

### 2.5. Characterization

#### 2.5.1. XRD

**Figure 2:**
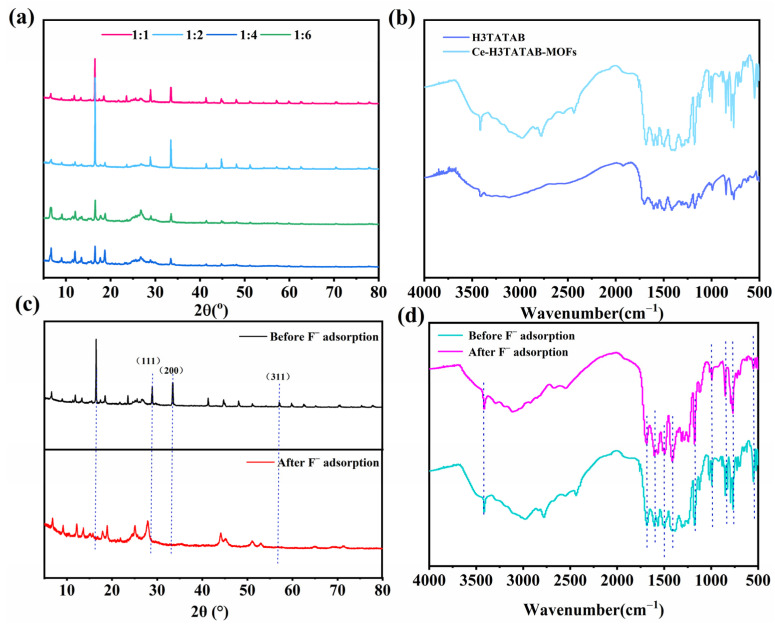
(**a**) XRD figures at different molar ratios; (**b**) FTIR spectra of H3TATAB and Ce-H3TATAB-MOF; (**c**) XRD figures of Ce-H3TATAB-MOFs before and after adsorption; (**d**) FTIR spectra of Ce-H3TATAB-MOFs before and after fluoride removal.

Ce-H3TATAB-MOFs was characterized via XRD to determine its crystallinity. As seen in Figure 2a from the XRD patterns of different molar ratios, the positions of the characteristic peaks of the materials under the conditions of each molar ratio remain the same, while the peak intensity is larger when the molar ratio is 1:1 and 1:2, and the crystallinity of its material is the highest. Combining with its adsorption capacity and removal efficiency of fluoride ions in solution, the molar ratio of 1:1 was chosen as the best molar ratio and was used for subsequent experiments. As seen in Figure 2c, the diffraction peaks of Ce-H3TATAB-MOFs were sharp, indicating that the material is highly crystalline [31]. The main crystalline peaks appeared at 2θ = 16.49°, 23.55°, 28.34°, 32.89°, and 55.77°. The XRD pattern recorded before F^−^ adsorption showed peaks of CeO_2_ at 2θ = 28.34°, 32.89°, and 55.77°, attributing to the (111), (200), and (311) crystalline planes, respectively [38,39]. After F^−^ attachment, the intensity of the peaks weakened and some of them disappeared, indicating that the metal was involved in the removal of F^−^, resulting in electrostatic interaction.

#### 2.5.2. FTIR

The FTIR spectra of Ce-H3TATAB-MOFs is displayed in Figure 2d. The asymmetric stretching vibrational peaks of -COOH in the ligand occurred at 1689 cm^−1^ and 1417 cm^−1^ (Figure 2b). However, after the reaction of the ligand with the metal Ce, the peaks of the C=O symmetric and symmetric stretching vibrations shifted to 1681 cm^−1^ and 1412 cm^−1^, red-shifted by 8 cm^–1^ and 5 cm^–1^, respectively, indicating that the Ce center was bound to the carboxyl oxygen atom in the ligand [40], and peaks at 1550 cm^−1^ that were owing to stretching vibrational peaks of the carbonyl and carboxylate anions of the MOF. The peaks of bending vibrational peaks of O-H presented at 1350 cm^–1^ and 500 cm^−1^; those at 1383 cm^−1^ were symmetric stretching vibrational peaks of C=O, and peaks at 1486 cm^−1^ were attributed to C=C and C=N bonds on the benzene ring and triazine [41]. In addition, the bending vibration peak of N–H and the symmetric stretching vibration peak of aromatic amine (C–N) appear at 1510–1525 cm^−1^. The peaks at 750 cm^−1^ indicate the presence of substituent groups on the benzene ring at the adjacent, para-, and inter-positions. The peak at 550 cm^−1^ is the characteristic peak of Ce–OH, and the peak at 3415 cm^−1^ is the stretching vibration peak of –OH. It can be revealed from the FTIR comparison before and after adsorption that the characteristic functional groups of the ligand were retained, indicating that Ce-H3TATAB-MOFs possesses good stability. Post adsorption, the spectral band frequencies of certain metal bonds were reduced owing to electrostatic interactions because exchangeable free OH^−^ in Ce-H3TATAB-MOFs were replaced with F^−^ via ion exchange [42] and the metal sites reacted with F^−^. After contact with the fluoride solution, the –OH peak at 3415 cm^−1^ remained in the same position but became sharper, indicating that the F^−^ and –OH on the surface of the adsorbent interacted to form an O–H···F bond [43].

#### 2.5.3. SEM and Energy-Dispersive Spectroscopy (EDS) Analyses

**Figure 3:**
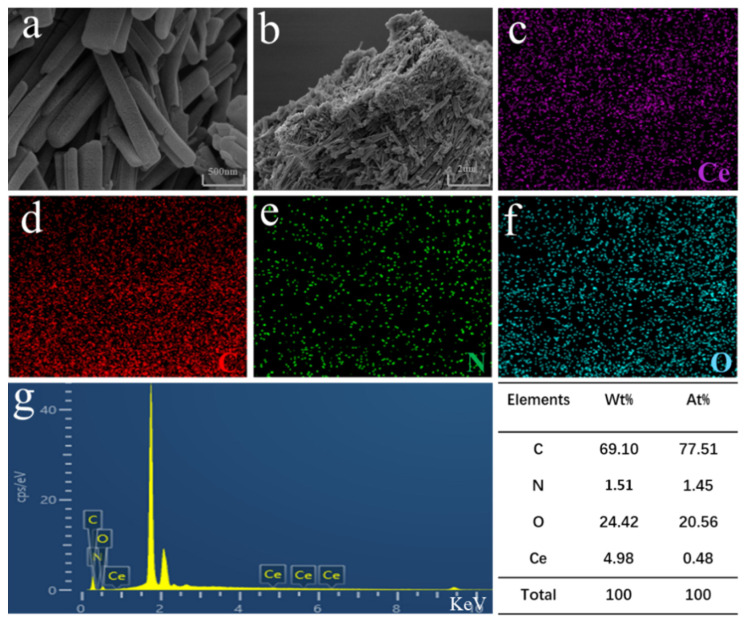
(**a**,**b**) SEM figures; (**c**–**g**) EDS mapping of Ce-H3TATAB-MOFs.

Representative SEM images of Ce-H3TATAB-MOFs are shown in Figure 3a,b, which reveal atypical rod-like structures with porous characteristics and uneven size distribution, where the distribution range of material size is 0.66~2.96 μm.

EDS mapping was performed to determine the elements present in Ce-H3TATAB-MOFs and the results are shown in Figure 3c–f. C, N, O, and Ce were unevenly distributed on the surface of the adsorbent, demonstrating that the active sites are abundant and uniformly distributed, which is beneficial to the adsorption of F^−^ from water.

#### 2.5.4. XPS

**Figure 4:**
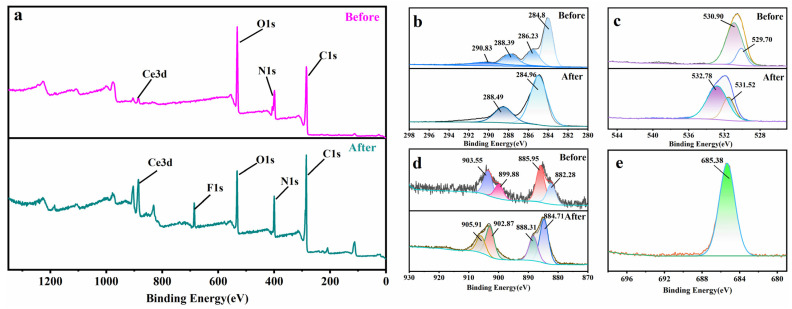
XPS spectra showing (**a**) survey spectra, and (**b**) C1s, (**c**) O1s, (**d**) Ce3d, and (**e**) F1s showing fine spectra of Ce-H3TATAB-MOFs.

XPS analysis was used to elucidate the adsorption mechanism by comparing the binding energy changes before and after adsorption. The results of the XPS analysis performed are shown in Figure 4. In the survey spectra (Figure 4a), the specific peak of F1s appeared at a binding energy of 685.38 eV after adsorption, demonstrating that fluoride was successfully captured by Ce-H3TATAB-MOFs. Figure 4b–e shows the fine spectra of C, O, Ce, and F. The C1s fine spectra before adsorption exhibited peaks at 284.8 eV, 286.23 eV, 288.39 eV, and 290.83 eV, which can be assigned to C–C, C–O, O–C=O, and –CH_2_–, respectively [25]. After adsorption, some of these peaks disappeared, and those at 284.8 and 288.39 eV shifted toward higher binding energy positions, indicating a reaction between –COOH and F^−^ on the adsorbent. In the O1s spectra, the characteristic peaks of lattice oxygen and surface active oxygen appeared at binding energies of 529.70 eV and 530.90 eV before attaching with the fluoride-containing wastewater [44]. After adsorption, the characteristic peak of lattice oxygen disappeared, demonstrating that a reaction between Ce and F^−^ occurred, generating a large amount of surface active oxygen that replaced the lattice oxygen (Ce–O). In the Ce3d spectra, two pairs of spin–orbit double peaks appeared for Ce3d_3/2_ and Ce3d_5/2_, respectively, with binding energies of 885.95 eV and 903.55 eV corresponding to the characteristic chemical state of Ce^3+^, and binding energies of 882.28 eV and 899.88 eV for Ce^4+^. Figure 4d shows that, after adsorption, the two pairs of spin–orbit bimodal peaks of Ce shifted toward higher binding energies, indicating that an electron transfer occurred between the Ce and F ions, forming a new complex.

#### 2.5.5. N_2_ Adsorption–Desorption Experiments

**Figure 5:**
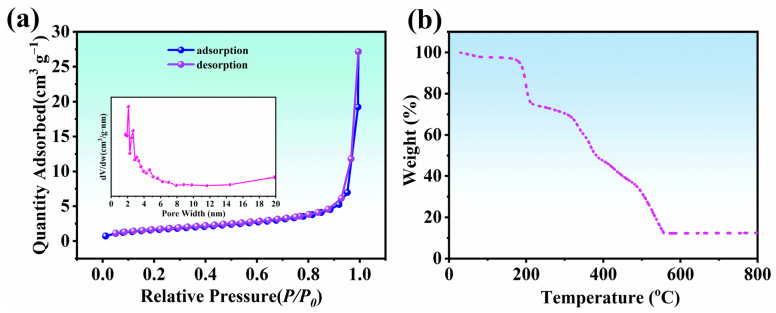
(**a**) N_2_ adsorption–desorption isotherms and (**b**) TGA curve of Ce-H3TATAB-MOFs.

The specific surface area and pore characteristics, which are key factors in determining the adsorption effect, were measured through N_2_ adsorption–desorption experiments. It can be observed in Figure 5a that the captured amount of N_2_ gradually increased with increasing relative pressure (*P*/*P_0_*) and then sharply increased when *P*/*P_0_* reached 0.8. According to the definition by the International Union of Pure and Applied Chemistry (IUPAC), this adsorption isotherm corresponds to a type-IV isotherm and was accompanied by a characteristic H3-type hysteresis loop typical of mesoporous structures [45].

#### 2.5.6. Thermogravimetric Analysis (TGA)

The thermal behavior was studied via TGA to investigate thermal stability [46]. As shown in Figure 5b, a series of weight losses occurred during the thermal decomposition process with the temperature change from 170–555 °C. The fact that the weight loss was negligible below 170 °C evidences the thermal stability of Ce-H3TATAB-MOFs up to this temperature. Between 170 °C and 300 °C, a weight loss of approximately 28% occurred owing to the release of bound water, free water, and DMF solvent [41]. A more significant weight loss of approximately 58% arose between 300 °C and 560 °C which may be ascribed to the decomposition of organic ligands. At this stage, the thermal stability of Ce-H3TATAB-MOFs decreased and its structure began to collapse. Above 560 °C, the Ce-H3TATAB-MOFs structure was close to complete decomposition and no further weight loss occurred. Therefore, the decomposed material can be deemed thermally stable and might have some practical application.

### 2.6. Adsorption Kinetics

In order to further comprehend the complete adsorption procedure, the experimental data were fitted by utilizing a quasi-first kinetic model, a quasi-second kinetic model, and an intra-particle diffusion model, respectively, with the three kinetic models expressed by Equations (1)–(3).

(1) $$\ln\left(q_{e}-q_{t}\right)=\ln q_{e}-K_{1}t$$

(2) $$\frac{t}{q_{t}}=\frac{1}{K_{2}q_{e}^{2}}+\frac{t}{q_{e}}$$

(3) $$q_{t}=K_{p}t^{\frac{1}{2}}+C$$

In these equations, *q_t_* and *q_e_* represent the sorption amount at time *t* (mg g^−1^) and equilibrium (mg g^−1^), respectively; *K*_1_ and *K*_2_ are the quasi-first adsorption and the quasi-second adsorption rate constant (min^−1^); *K_p_* is the intra-particle diffusion rate constant (mg (g·min^1/2^)^−1^); and *C* is a constant.

**Figure 6:**
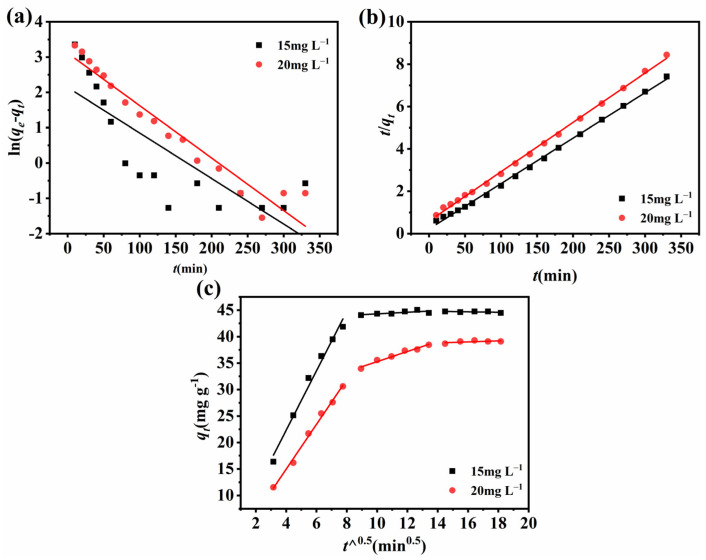
(**a**) quasi-first-order model, (**b**) quasi-second-order model, and (**c**) intra-particle diffusion model for defluoridation of Ce-H3TATAB-MOFs.

The fitted results are displayed in Figure 6 and the parameters of each model are shown in Table 1 and Table 2. The coefficient of determination R^2^ indicates the correlation between the experimental data and the kinetic model where the higher the R^2^ value, the better the correlation [47]. The fitted parameters and the model fit plots reveal that the kinetic is more in accordance with the quasi-second kinetic model. This indicates that the rate-determining step is dominated by chemisorption and that the adsorption process mainly involves electron sharing or electron exchange between the sorbent and the adsorbate, resulting in valence forces [48].

### 2.7. Thermodynamic and Isothermal Research on Adsorption

Investigating the thermodynamics and relevant parameters is essential for understanding the adsorption procedure. The thermodynamic parameters of Δ*G^o^*, Δ*H^o^*, and Δ*S^o^* were calculated here using Equations (4)–(6), respectively [29]:

(4) $$K_{d}=\frac{q_{e}}{C_{e}}$$

(5) $$\Delta G^{o}=\Delta H^{o}-T\times \Delta S^{o}$$

(6) $$\ln K_{d}=-\frac{\Delta H^{o}}{RT}+\frac{\Delta S^{o}}{R}$$

where $K_{d}$ is the dispersion coefficient, Δ*G^o^*, Δ*H^o^*, and Δ*S^o^* are the Gibbs free energy (kJ mol^−1^), enthalpy change (kJ mol^−1^), and entropy change (J (mol·K) ^−1^), respectively, *T* represents the temperature (K), and *R* is the gas constant with a value of 8.314 J (mol·K) ^−1^.

**Figure 7:**
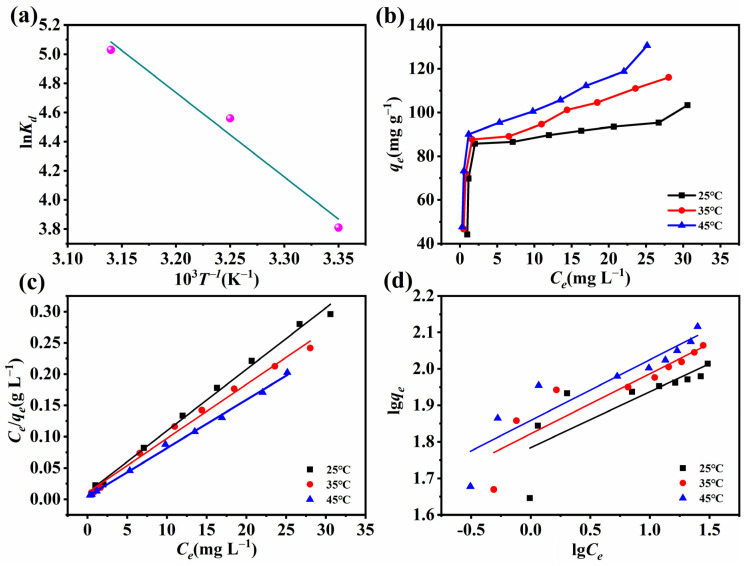
(**a**) Plotted ln*K_d_* against 10^3^ *T*^−1^, (**b**) adsorption thermodynamics, (**c**) Langmuir model, and (**d**) Freundlich model.

Table 3 shows the calculation results for the above parameters. The enthalpy is positive, suggesting that the entire sorption process is a heat absorption procedure. Moreover, in conjunction with Figure 7b, the results indicate that increasing the temperature is conducive to adsorption. The positive entropy suggests that the adsorption process becomes progressively more complex, and the negative Gibbs free energy, the absolute value of which increases with increasing temperature, manifests that high temperature can increase the driving force of the adsorption process, and physical sorption on Ce-H3TATAB-MOFs is accompanied by chemisorption [49].

Adsorption isotherm studies [50] are also important in understanding the process of F^−^ removal, and are crucial for explaining the interaction between adsorbate and adsorbent in order to optimize the adsorbent used. Herein, using the Langmuir and Freundlich models to fit the experimental data, we evaluate the thermodynamic adsorption process in conjunction with the obtained correlation coefficients [51].

The Langmuir model is defined by non-linear Equation (7) and linear Equation (8) as follows:

(7) $$q_{e}=\frac{K_{L}q_{m}C_{e}}{1+K_{L}C_{e}}$$

(8) $$\frac{C_{e}}{q_{e}}=\frac{1}{K_{L}q_{m}}+\frac{C_{e}}{q_{m}}$$

The Freundlich model is expressed by non-linear Equation (9) and linear Equation (10):

(9) $$q_{e}=K_{f}C_{e}^{\frac{1}{n}}$$

(10) $$\mathrm{lg}q_{e}=\mathrm{lg}K_{f}+\frac{1}{n}\mathrm{lg}C_{e}$$

where *q_e_* (mg g^−1^) and *q_m_* (mg g^−1^) are the equilibrium sorption capacity and maximal sorption capacity, respectively; *Ce* is the F^−^ concentration at equilibrium (mg L^−1^); *K_L_* is the adsorption equilibrium constant (L mg^−1^); *K_f_* is the adsorption constant reflecting the amount of adsorption (L g^−1^); and n is the adsorption constant reflecting the intensity of sorption.

The above-described experiment was repeated with the temperatures of 298–318 K, and the results of fitting each model are presented in Figure 7 and Table 4. In the case of the Langmuir model, the results fitted well with a correlation coefficient (R^2^) of 0.9974, which is larger than that achieved with the Freundlich model. Therefore, the Langmuir model is deemed more proper for describing the thermodynamic sorption process, implying that the fluoride removal procedure is dominated by monolayer sorption.

### 2.8. Field Application

The applicability of Ce-H3TATAB-MOFs on a practical level was evaluated with a water sample taken from a nearby fluoride-endemic area. A sample solution of 50 mL contained 0.01 g of Ce-H3TATAB-MOFs in polypropylene flasks was shaken at 150 rpm in a thermostatic shaker at 25 °C for 12 h. In the fluoride-containing wastewater, the fluoride concentration was observed as 11.3 mg L^−1^. After adsorption, the fluoride concentration of the field water reduced from 11.3 to 0.56 mg L^−1^, below 1.0 mg g^−1^ (shown in Table 5). Additionally, the Ce-H3TATAB-MOFs reduced and controlled other parameters of water quality (PWQ) such as COD.

### 2.9. Performance Comparation of Ce-Based Adsorbents for Fluoride Removal

A comparative investigation was carried out to studied the defluoridation performance of Ce-H3TATB-MOFs. The adsorption capacity of F^−^ of the prepared adsorbent was compared with previously reported studies (Table 6). Apparently, the sorption capacity of Ce-H3TATAB-MOFs was superior, revealing that the present adsorbent has the potential to removal fluoride from aqueous solutions.

### 2.10. Mechanism

**Figure 8:**
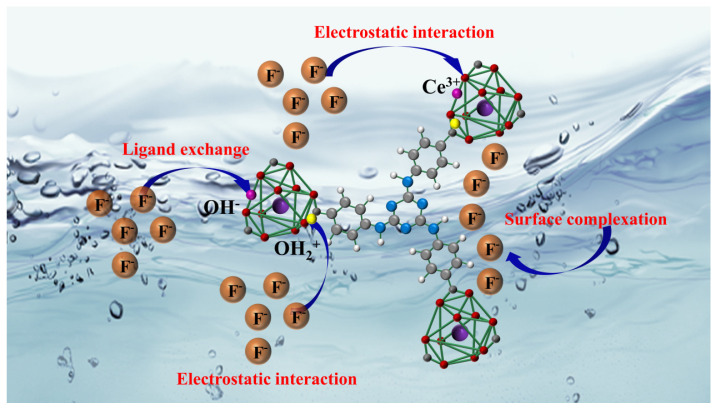
Mechanism schematic of Ce-H3TATAB-MOFs for defluoridation.

Typically, studying the adsorption mechanism requires determination of the properties of the adsorbent (including surface properties, charge condition, crystalline structure, and chemical bonding characteristics), as well as adsorption kinetics, adsorption thermodynamics, and specific interactions between the adsorbent and F^−^. The mechanism schematic of Ce-H3TATAB-MOFs for defluoridation was shown in Figure 8. Herein, the characterization analysis, adsorption thermodynamics, and kinetics indicate that the –OH groups on the adsorbent and F^−^ in the solution form a Ce–F complex via ligand exchange. Moreover, as a hard acid, Ce^3+^ can easily react with F^−^ in solution through electrostatic adsorption and surface complexation, thus realizing F^−^ removal.

## 3. Materials and Methods

### 3.1. Reagents

AR grade cerium nitrate (Ce(NO_3_)_3_·6H_2_O; Shanghai Maclean Biochemical Technology Co., Ltd., Shanghai, China), AR grade N,N-dimethylformamide (DMF; Xilong Chemical Co., Ltd., Guangzhou, China), 4,4′,4″-((1,3,5-triazine-2,4,6-triyl) tris (azanediyl)) tribenzoic acid (H3TATAB) (Shanghai Kai Shu Chemical Technology Co., Ltd., Shanghai, China), 36~38% hydrochloric acid (HCl; Xilong Chemical Co., Ltd.), and AR grade sodium hydroxide (NaOH; Sinopharm Chemical Reagent Co., Ltd., Shanghai, China) were considered as received. All experiments were conducted using ultrapure water.

### 3.2. Preparation of Ce-H3TATAB-MOFs

The preparation of Ce-H3TATAB-MOFs was followed according to “Microporous La–Metal–Organic Framework (MOF) with Large Surface Area” [52]. A mixture of DMF (3 mL), anhydrous ethanol (5 mL), distilled water (2 mL), and ethylenediamine (0.048 mL) was shaken in a 20 mL reagent bottle and set aside. According to the metal/ligand molar ratio of 1:1, 0.038 g of Ce(NO_3_)_3_·6H_2_O and 0.0426 g of H3TATAB were added to the solution, and after mixing evenly, 0.8 mL of 16 mol L^−1^ nitric acid (HNO_3_) was added dropwise. The reagent bottle was put in a drying oven at 100 °C for 72 h. Subsequently, the reagent bottle was cooled to room temperature, the final solution was removed, washed with anhydrous ethanol, and filtered. The resulting solid material was dried in an oven at 60 °C for 12 h to afford the adsorbent, which was labeled Ce-H3TATAB-MOFs.

### 3.3. Material Characterization

The morphology of the material was viewed via scanning electron microscopy (SEM) using a Tescan MIRA LMS microscope (Brno, Czech Republic). X-ray diffraction (XRD) was performed to study the crystal structure of the adsorbent using a Rigaku SmartLab SE X-ray diffractometer (Tokyo, Japan) with a scanning angle from 5° to 80° and a scanning speed of 5° min^−1^. The surface elements of the sample and the functional group distribution of the adsorbent were investigated via X-ray photoelectron spectroscopy (XPS) using a Thermo Fisher ESCALAB 250Xi spectrometer (Waltham, MA, USA). The vacuum of the analysis chamber was 4 × l0^−9^ mbar, the excitation source was Al k (*hv* = 1486.6 eV), the operating voltage was 14.6 kV, the filament current was 13.5 mA, and the signal was accumulated for 20 cycles. The test through-energy (Passing-Energy) was 20 eV in steps of 0.1 eV.

### 3.4. Batch Experiments

Batch adsorption experiments were conducted to investigate the defluoridation performance of Ce-H3TATAB-MOFs.

In brief, fluoride solutions with several initial concentrations (10–50 mg L^−1^) were placed in a 50 mL volumetric flask, the pH was equal to 4, the dosage of Ce-H3TATAB-MOFs was 0.01 g, and the mixture was rocked at 25 °C for 12 h. Subsequently, it was filtered and the supernatant was extracted to measure its potential.

To study adsorption kinetics, the fluoride concentration was set at 10, 15, and 20 mg L^−1^, respectively, the reaction temperature was 25 °C, and the pH of the solution was 4. The amount of Ce-H3TATAB-MOFs used was 0.01 g. Samples were extracted at different time intervals. Specifically, the sampling was performed every 10 min for the first 1h, every 20 min for the next 2–3 h, and every 1h for the following 4–6 h.

For the adsorption thermodynamic experiments, F^−^ solutions with varied concentrations (10–50 mg L^−1^) were placed in a 50 mL plastic tube, the pH of the solution was controlled at 4, then added to the Ce-H3TATAB-MOFs (0.01 g), and the reaction was conducted at three temperatures of 298 K, 308 K, and 318 K for 12 h. Subsequently, the solution was left to filter and the supernatant was extracted to measure its potential. The adsorption capacity and sorption efficiency were estimated using Equations (11) and (12) as follows [53].

Adsorption capacity:

(11) $$q_{e}=\frac{(C_{0}-C_{e})V}{m}$$

Sorption efficiency:

(12) $$\eta =\frac{C_{0}-C_{e}}{C_{0}}\times 100\%$$

To discuss the influence of pH on the elimination of F^−^, the pH of the solution was regulated at 3–10 using HCl and NaOH. After adding 0.01 g of Ce-H3TATAB-MOFs to 50 mL of a 20 mg L^−1^ F^−^ solution, the reaction was performed at a constant temperature under shaking for 12 h. The F^−^ concentration was measured.

In the interference experiments, Cl^−^, NO_3_^−^, CO_3_^2−^, SO_4_^2−^, PO_4_^3−^, and H_2_PO_4_^−^ were added to a fluoride-containing solution with a concentration of 20 mg L^−1^. The solution pH was controlled at 4.0 and the dose of Ce-H3TATAB-MOFs was 0.01 g. The same procedure as that described for studying the impact of pH was performed.

In the above experiments, both the concentration of fluoride ions in the supernatant and pH were determined using a Shanghai Yidian Scientific Instruments Co., Ltd. (Shanghai, China). PF-2(01) type fluoride ion electrode according to the National Standard of the People’s Republic of China (GB 7484-87), and a PHSJ-4A type pH meter, respectively.

## 4. Conclusions

The H3TATAB-containing Ce-H3TATAB-MOFs prepared in this study exhibited outstanding adsorption selectivity for F^−^ in wastewater with a wide pH range of application (3–10). Further, it attained a satisfactory removal efficiency of 76.57% under strongly alkaline conditions (pH 10), resulting in a F^−^ concentration (<1.0 mg L^−1^) below the WHO threshold of 1.5 mg L^−1^. Kinetic and thermodynamic experiments showed that the quasi-second order model and the Langmuir model fitted the experimental data well, demonstrating that the adsorption process was controlled by monolayer chemisorption. Interference experiments show that only PO_4_^3−^ and H_2_PO_4_^−^ mitigated the removal efficiency, whereas other competing anions had no adverse effect on F^−^ removal. Overall, the characterization suggests that Ce-H3TATAB-MOFs is a mesoporous material with prominent adsorption properties, and the F^−^ removal mechanism involves ligand exchange, electrostatic adsorption, and surface complexations that form Ce–F endo-complexes.

## Figures and Tables

**Table 1:** Fitted parameters of the quasi-first order and quasi-second order models.

Models	*C*_0_ (mg L^−1^)	*K*	*q_e_*	R^2^
quasi-first order	15	0.0129	8.5071	0.6754
	20	0.0149	22.5042	0.9399
quasi-second order	15	0.0021	46.5100	0.9983
	20	0.0009	42.9184	0.9985

**Table 2:** Fitted date of intra-particle diffusion model.

*C*_0_ (mg L^−1^)	Equation	R^2^
15	*y* = −0.1611 + 5.615*x*	0.9843
	*y* = 42.7577 + 0.1544*x*	0.5380
	*y* = 45.4148 − 0.0449*x*	0.2672
20	*y* = −1.9843 + 4. 2339*x*	0.9941
	*y* = 25.7361 + 0. 9544*x*	0.9729
	*y* = 37.4474 + 0. 0973*x*	0.3633

**Table 3:** Thermodynamic parameters of adsorption for fluoride removal.

*T* (K)	Δ*G^o^* (kJ mol^−1^)	Δ*H^o^* (kJ mol^−1^)	Δ*S^o^* (J (mol·K)^−1^)
298	−9.5039	48.0881	193.2618
308	−11.4365		
318	−13.3692		

**Table 4:** Fitted parameters of the Langmuir and Freundlich model.

*T* (K)	Langmuir			Freundlich		
	*q_m_* (mg g^−1^)	*K_L_* (L mg^−1^)	R^2^	*K_f_* (L g^−1^)	n	*R* ^2^
298	101.6260	0.9128	0.9950	72.1855	6.0168	0.8494
308	115.7407	0.8105	0.9924	66.3315	6.0683	0.8205
318	129.7017	1.7058	0.9974	60.7813	6.5134	0.6650

**Table 5:** Field trail results of Ce-H3TATAB-MOFs.

Water Quality Parameters	Before Adsorption	After Treatment
F^–^ (mg L^–1^)	11.3	0.56
pH	3.67	3.95
COD (mg L^–1^)	125	74

**Table 6:** Comparison of fluoride sorption capacity with previously reported adsorbents.

Adsorbents	pH	*q_m_* (mg g^–1^)	References
HAP-Ce-BTC-MOFs	6	4.46	[34]
Ce-ABDC	6–7	4.88	[38]
Ce-BDC	6–7	4.91	[38]
Ce-UIO-66 MOF	3	66.1	[44]
Ce-bpdc	7	45.5	[45]
Ce-AlOOH	3	62.77	[46]
Ce@BTC MOFs	7.49	4.94	[47]
Ce-BTC	3–9	70.7	[48]
Ce-fum	4	78.94	[49]
Ce-H3TATAB-MOFs	4	129.7	This study

## Data Availability

The data are included within the article.
